# Dendritic cells and macrophages neurally hard-wired in the lymph node

**DOI:** 10.1038/srep16866

**Published:** 2015-11-19

**Authors:** Clemens Wülfing, Hauke S. Günther

**Affiliations:** 1 Group for interdisciplinary neurobiology and immunology, Biozentrum Grindel, University of Hamburg.

## Abstract

The neural hard-wired pathways in which the lymphoid organs are innervated by the nervous system is of special interest with respect to suggested afferent and sensory systems informing the central nervous system about the status of the immune system. Until today efferent also like afferent innervation seem to be unspecific, targeting many types of cells by affecting many cells at the same time. We for the first time show that antigen presenting cells (APC) are abundantly innervated in the T-cell enriched area, the subsinoidal layer and the cortical extrafollicular zone of lymph nodes in rats by a mesh of filamentous neurofilament positive structures originating from single nerve fibers and covering each single APC similar to a glass fishing float, so that we termed them “wired” APC (wAPC). These wAPC also found in humans seem to be restricted to the cell body, not to follow membranous extensions, they may be dynamic and receptive as MAP2 is expressed and axonal growth cones can be detected and they probably lack vesicular activity through missing synaptophysin expression. The specific innervation targeting single cells which show a distribution divided in several areas in one lymph node suggests a form of topographically organized afferent sensory system.

The bi-directional communication between the central nervous system (CNS) and the immune system is an essential part of research in the field of psychoneuroimmunology, a scientific discipline founded nearly 40 years ago by Robert Ader and Nicolas Cohen^1^. Beside the long known humoral pathways, in the last years the focus on neural – or so called hard wired - pathways has emerged. And although in mammalian there is a large amount of knowledge about the sympathetic system and the efferent innervation of primary and secondary lymphoid organs, relatively little is known about afferent pathways or the role of the parasympathetic system^2^,^3^,^4^.

Taking a closer look on the innervation of secondary lymphoid organs with focus on lymph nodes many results have been achieved decades ago. Today with the fact of autonomic innervation of lymphoid organs still being absent in most standard textbooks, sympathetic efferent innervation is established by methods of staining for catecholamines and their cotransmitters like neuropetides, silver impregnation, neurectomy or tracing experiments^5^,^6^,^7^,^8^. Until today only a handful of papers touched the possible afferent pathways, mostly by functional and morphological neuroanatomy or vagotomy experiments respectively. Interestingly, no discrimination between viscera draining and skin draining lymph nodes regarding possible afferent innervation has been made, as the latter ones are probably no target of the vagus nerve. Anyhow, in both pathways only wide or close junctional neurotransmission has been suggested, so that - in a general opinion - the influence of the CNS on functions in the immune system follows the principles of volume transmission, with innervation not being restricted to the endothel, but also targeting cells in the parenchyma. Regarding the sensible vagus-related innervation of the lymph node, a role for neuropeptide containing nerve fibers has been suggested as has been a sensory system in which immune cells report the immune status to the CNS, but no morphological suggestion has been made until today^9^,^10^,^11^,^12^.

Turning the eye on the lymph node cell population as the target of efferent like also afferent innervation it has been proved, that beside the known endothelial innervation most of the nerve fibers reaching the surrounding parenchyma avoid the B-cell follicles^6^,^12^,^13^. So except the latter region nerve fibers have been found in all three anatomical defined areas, cortex, paracortex and medulla, taking into consideration that also alternative models exists. Beside the lymphocyte cell population, which has most times supposed to be the candidate for the bi-directional communication, the organ is built up of stromal cells, cells of the so called mononuclear phagocytic system and the dendritic cell (DC) population with some overlaps in their characterization and sometimes no clearly visible distinction^14^. So there is not only an ongoing discussion about the relationship between macrophages and DC^15^ (Conference Macrophages and DC reunited, Canada 2015), it still is debated, what makes up the differences between the various described DC subpopulations and their origin and function. So the actual known DC can be antigen-librarys, can be migratory and resident, can obtain antigen in the periphery or soluble antigens in conduits for presenting it to lymphocytes and they even can be a part of and also control the recently discovered conduit system of the lymph node beside fibroblastic and marginal reticular cells^16^,^17^,^18^,^19^,^20^. And with macrophages closing more and more gaps to the functions of DC, the flexibility of these two antigen presenting cell (APC) populations seem to be infinite, so they also may be ideal candidates for communicating with the nervous system?

In this work, we not only found that there is a tremendous amount of short branched axonal structures in the T-cell enriched area, we also show for the first time, that single cells with APC character are “wired” to single nerve fibers by a dense and closely associated meshwork of neural structures covering the cell body. And because of their first characterization done here, we propose that this cells could function as a dynamic afferent sensory system for the CNS to get detailed information on single cell level in a topographically organized manner about ongoing immune responses.

## Results

**Figure 1:**
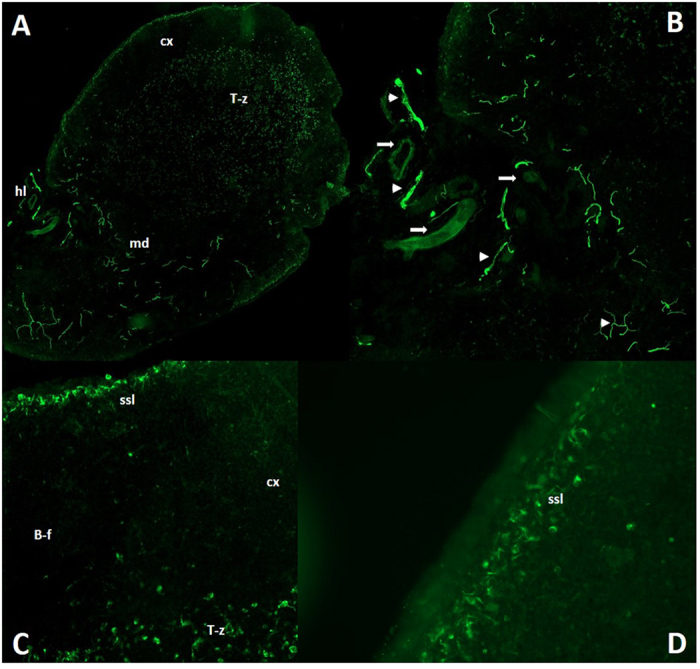
Nerval structures are manifold throughout the lymph node. Superficial cervical lymph nodes of DS rats stained with monoclonal anti-neurofilament (green). Image (**A**) shows an overview (20 fold) of the lymph node and Image (**B**,**C**) are magnifications of sections of Image (**A**) (also 20 fold). The intensive and varying staining patterns of the different anatomical areas of the node can be seen in (**A**). Whereas Image (**B**) shows the axonal structures coming as a thick nerve from the hilus, splitting up to finer fibers along their way through the medulla (white arrowheads) and being clearly separated from the also stained fine nerve plexus following the vasculature (white arrows). Image (**C**) and also image (**D**) (40 fold/from another node) show the intensive innervation of the subsinoidal layer and the missing innervation of the B-cell follicles. wAPC could be seen in all images in the T-cell enriched zone and–to a lesser extent–in the subsinoidal layer. Cx–cortex/T-z–T-cell enriched zone/md–medulla/hl–hilus/ssl–subsinoidal layer/B-f–B-cell follicle.

**Figure 2:**
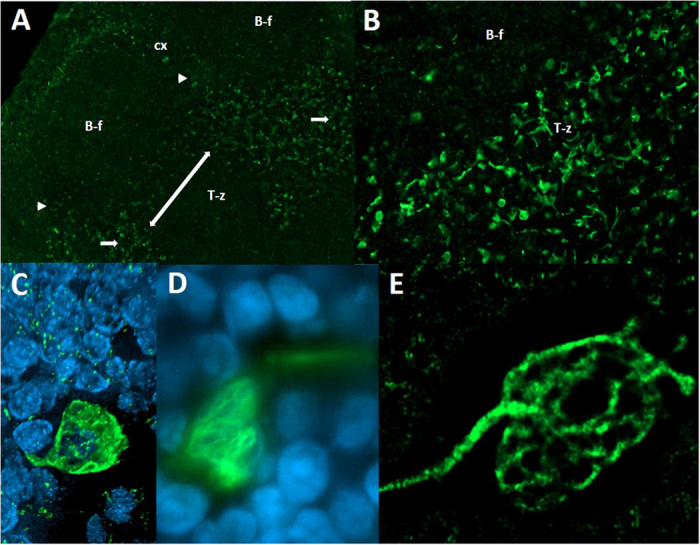
Uncovering the wAPC. Superficial cervical lymph nodes of DS rats stained with monoclonal anti-neurofilament (green) and DAPI (blue). Image (**A**) shows two B-cell follicles in the cortex region of the lymph node with a part of the underlying T-cell enriched zone. The wAPC are located mainly in the T-cell enriched zone, but continue also in the interfollicular area (white arrowheads) by excluding the follicles. A clear discontinuity between two wAPC areas (white arrows) in the T-cell enriched zone could be seen (white double arrow-ended line). Image (**B**) shows the dense innervation of wAPC in the T-cell enriched region, with single cells reached by single nerve fibers (20 fold and 40 fold respectively). Image (**C**) as a 3D reconstruction (z-stack) shows one single wAPC reached by one nerve fiber, and illustrating the dense mesh around the cell staining with DAPI for a large nucleus (100 fold). Image (**D**,**E**) show the filamentary structure of the mesh around the wAPC resembling a “glass fishing float” (100 fold). Cx – cortex/T-z – T-cell enriched zone/B-f – B-cell follicle.

**Figure 3:**
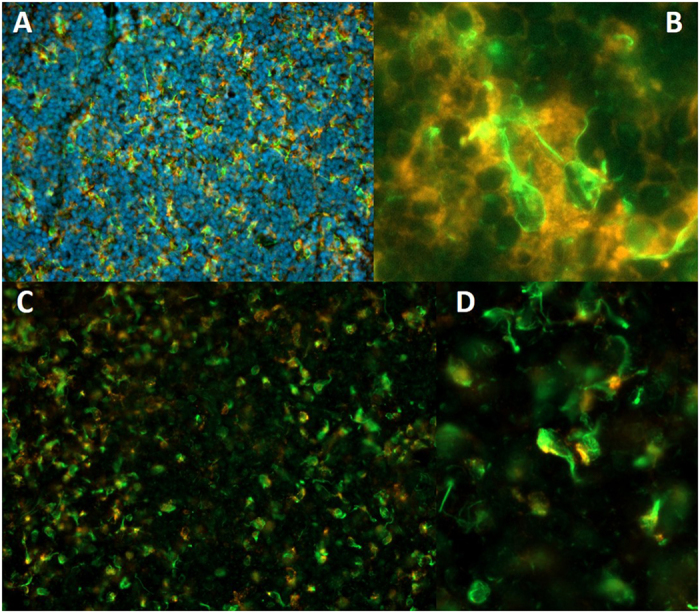
All wAPC seem to be APC and express MHC II and the majority also express a macrophage marker. Superficial cervical lymph nodes of DS rats stained with monoclonal anti neurofilament (green) and monoclonal anti-MHC II (orange) in Image (**A**,**B**), and anti neurofilament (green) and monoclonal anti-CD68 (orange) in image (**C**,**D**). Image (**A**) shows the wAPC in the T-cell enriched zone marked by neurofilament staining and the colocalization with MHC II (40 fold). Image (**B**) gives a closer look to the remarkable MHC II expression around two wAPC which are both reached by single nerve fibers and illustrates the neural mesh restricted to the cell body not covering membranous extensions like dendrites (100 fold). Image (**C**) shows the wAPC in the T-cell enriched zone marked by neurofilament staining and the colocalization with CD68. Clearly, the macrophage related marker CD68 appears in more than ¾ of all wAPC. Image (**D**) gives a closer look on the intracellular lysosomal CD68 signal. (Both 40 fold, D is a magnification of a section of image (**C**)).

**Figure 4:**
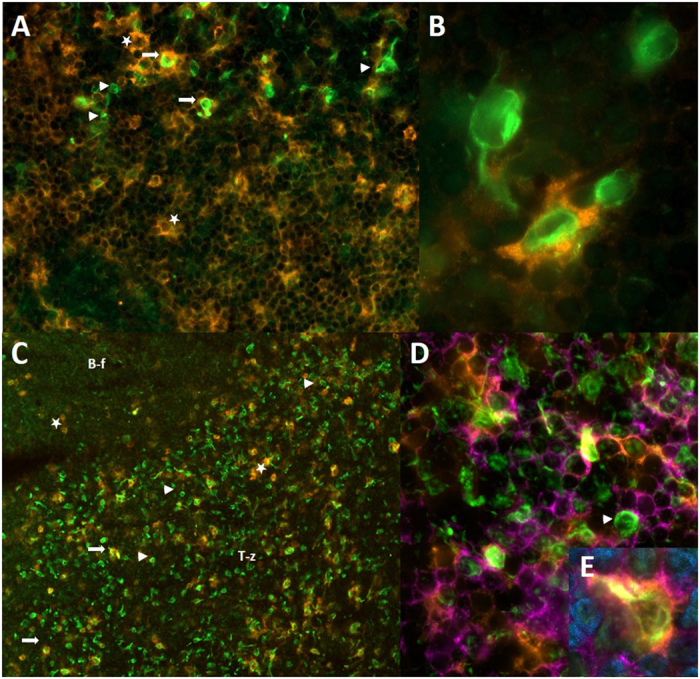
Every wAPC expresses different APC surface markers. Superficial cervical lymph nodes of DS rats stained with monoclonal anti neurofilament (green) and monoclonal anti-CD11b/c (orange) in Image (**A**) (40 fold) and (**B**) (100 fold), monoclonal anti-CD103 (orange) in Image (**C**) (20 fold) and monoclonal anti-CD11b/c (orange) and monoclonal anti-CD4 (purple) in Image (**D**) (100 fold) and E (100 fold). Image (**A**) points out some wAPC expressing CD11b/c (white arrows) and some not (white arrowheads), by also showing some CD11b/c positive cells without neurofilament staining (white asterixes). Image (**B**) shows two wAPC close together, one expressing CD11b/c and the other being negative for that marker and illustrates the neural mesh restricted to the cell body not covering membranous extensions like dendrites. Image (**C**) as another example shows some wAPC being positive for CD103 (white arrows), some being negative (white arrowheads) and some cells being positive for CD103 which have no neurofilament staining (white asterixes). Image (**D**) displays the very heterogenous expression of the surface markers CD11b/c and CD4 with some wAPC expressing none (white arrowhead) and others all three of them (Image E of the same area). T-z – T-cell enriched zone/B-f – B-cell follicle.

Our first approach was based on the fact that it has been very difficult to stain neural structures in the lymph node in general, whereas almost every experimental trial has used fixated tissue obtained by various methods. We thought that it would be best to use tissue as much untreated as possible, so we took freshly prepared cryo-sections of snap frozen lymph nodes of the rat for staining with anti-neurofilament. The stunning results showed even in the first section a manifold of neural structures and an intensive innervation of the lymph node parenchyma beside the known innervation of the vasculature. Thick and branched axons were visible mainly in the medullary part of the lymph node, which appeared to be different of the fine fibers staining around the blood vessels (Fig. 1 image A,B). A closer look at the tissue parts reached by nerval structures revealed beside innumerable nerve-fibers also single cells which are enclosed with a very thin and filamentary mesh that showed neurofilament staining, and because of their following characterization with APC-related markers together with their connection to single nerve fibers we would like to refer to that cells in the following with the term “wired-antigen presenting cells” or wAPC. These wAPC like the countless nerve-fibers were located in deliminated areas above the medulla, neither in B-cell follicles nor close to high endothelial venules, and mainly in the T-cell enriched region, which we proved by anti-CD3 and anti-endothelial (RECA-1)-staining (Fig. 1 image A,B, Fig. 2 image A,B and supplement Figure S1). Some wAPC seemed also to be dispersed in the subsinoidal layer, but nerve fibers which seemed not to be “hard-wired” to cells dominated in this region (Fig. 1 image C,D). The arrangement of that wAPC containing areas in the T-cell enriched region did not show a continuous course across the lymph node parenchyma as someone would expect from the concept of a continuous paracortex, the areas are rather clearly discontinuous and most times center below a cortical region which was in between two B-cell follicles and also contained some interspersed wAPC (Fig. 2 image A). The wAPC were always polymorph cells with much cytoplasm and a large light staining nucleus and the neurofilament positive mesh originating from single nerve fibers was regularly covering the cell body very closely similar to a glass fishing float as shown in Fig. 2 image C–E. This round and mostly oval structure of the neural mesh around the wAPC cell body seem not to follow any cellular membranous extension (Fig. 3 image B and Fig. 4 image B), which could be expected because of the dendrites normally formed by DC and to a lesser extent also by macrophages.

Next we wanted to know if the wAPC are potentially dynamic and possibly receptive, and therefore associated with other neural markers like MAP2, which is more related to neurogenesis and indicating dendritic and receptive structures in the CNS^21^, and an antibody for marking axonal growth cones, only to get a first hint. We found positive results both for MAP2 and the anti-growth cone 2G13p, the results are shown in supplement Figure S2. MAP2 shows a granular and dotted signal whereas neurofilament stains for the typical filamentous structures. Both signals turned out to be colocalized around the wAPC, with the MAP2 signal seeming to surround the neurofilament signal and cells only with MAP2 expression could been found, but nearly no neurofilament positive cell without MAP2 could be detected. Axonal structures located in the medullary region did not show any MAP2 expression. Axonal growth cones are stained in between the wAPC and often show partly coexpression with neurofilament in their proximal part. Also here, no medullary axonal growth cone could be detected, and also the B-cell follicles and subsinoidal layer were devoid of that structure.

It was an obvious question that followed, namely which type of immune cell would be the candidate for representing the wAPC reached by that neural structures. Because of the above described morphology we suggest that APC should be a good candidate, so we decided to use DC and macrophage markers that have turned out to be successful in rats, for probing that hypothesis, namely MHC II, CD4, CD11b, CD11c, CD103, CD68 and SIRPα^22^,^23^,^24^,^25^, which we stained in different combinations. Four results could be stated: First, all wAPC express high levels of MHC II, which could nicely be seen in the colocalization in Fig. 3 image A,B. Second, except MHC II and SIRPα, there is no marker positive for nearly all of the wAPC, it seems as if every wAPC expresses its own subset of CD4, CD11b, CD11c, and CD103 as outlined in Fig. 4, with some wAPC being negative for all markers and many other cells positive for one or more markers but without any neural contact. Another striking feature noticed was the fact that third, more than ¾ of the wAPC express the more macrophage related marker CD68 (Fig. 3 image C,D). Proceeding with that, the picture completely changed when staining for SIRPα, so we fourth found not only a nearly complete colocalisation between neurofilament positive and SIRPα positive wAPC, even the staining pattern turned out to be identical as if the nerval structures express SIRPα themselves (supplement Figure S3). As CD90 was also used in defining rat APC and shows a similar tissue representation like SIRPα namely in neurons like in immune cells we tested its expression with the wAPC, but with CD90, there was no colocalization detectable. Beside that remarking results, we wanted to exclude the possibility that also other stromal cells could be candidates for the wAPC, so we used the markers Podoplanin and CD31 for the recently discovered stromal cells and also S100 used by another group for marking another cell type reached by nerval structures^8^,^18^,^19^,^20^. All this markers turned out to be negative (data not shown) underlining the characterization of the wAPC as being an innervated APC.

The last question we wanted to ask at this stage was, as if we can find any first hint for the origin of the wAPC innervation. As the complete work to do for elucidating the CNS pathways (current work is in progress) would be beyond the scope of this late-breaking but short report, we decided to test for the sympathetic origin^3^,^5^ with two approaches: First we checked for synaptical transmission. As with that type of transmission vesicles are used, we stained for synaptophysin. The results show clearly that there is no synaptophysin expression located at the wAPC, whereas we found a known expression with the medullary and vasculature related neural structures innervating the endothel. Second, we did an anterograde tracing by injecting Fluoro-Ruby into the ganglion cervicale superius for tracking the related sympathetic efferences and subsequently stained for neurofilament. According to that preliminary data, the tracing signals we identified, seemed to be not colocalized with the wAPC (supplement Figure S4).

It should be noticed that at the time of this publication, we already confirmed the existence of wAPC in other vertebrates, including humans and astonishingly also in T-cell deficient mice (supplement Figure S5).

## Discussion

With this new type of innervation morphology, where the wAPC described here were closely covered by a neural meshwork, many questions arise.

As we have shown, there is formidable evidence, that the wAPC are in fact antigen presenting cells because of their expression of many typical markers of dendritic cells and also macrophages. On the other side we were not able to define a specific subset of markers which characterizes all of the wAPC and with just CD68 as a typical macrophage marker marking the majority of wAPC that also express DC markers and that also anatomically should be mostly DC in that T-cell enriched region, the question about the differences between macrophages and DC raises again. One result is in need of an explanation through further investigations: the complete colocalisation between the neurofilament and the SIRPα staining, namely which type of cell does express SIRPα, the wAPC themselves or the endings of the nerve fibers covering them, hereby taking into consideration that SIRPα expression has already been shown in neurons^26^. The origin of the SIRPα expression is currently under investigation.

Looking at the arrangement of the wAPC areas and their discontinuous course through the paracortical and T-cell enriched region, it points out a concept that has almost fallen by the wayside: The concept of the deep cortex first suggested by Sainte Marie and colleagues^27^. Therefore we suggest that every wAPC-area corresponds to a deep cortex center and the interspersed wAPC in the cortical regions between B-cell follicles would therefore be located in the extrafollicular zone being one part of the peripheral cortex.

So with the characterization of the wAPC as an antigen presenting cell neurally hard-wired and grouped in separated areas in the T-cell enriched region one very important question is whether the wAPC function as an efferent or afferent system of the central nervous system.

To get first hints, we did the synaptophysin staining and the tracing experiments outlined above. As the tracing experiments strongly emphasized a non-sympathetic origin, also the missing synaptophysin expression nearly excluded a noradrenergic innervation by sympathetic nerve fibers, indicating a different origin of the wAPC innervation and enhancing an afferent and therefore possibly sensory role of that cells.

Beyond that, the fact that synaptophysin can be found in a ubiquitous manner in nearly all vesicular synaptic transmission, as these molecule can also be used for total synapse quantification independent from the quality of its transmitters, can give further hints. So the absence of synaptophysin expression in the area of the neural meshwork surrounding the wAPC does not only point out a missing sympathetic catecholamine release, it also indicates, that this new nerve- to immune-cell contact we found lacks synaptic vesicular activity in general, therefore probably also all normally existing cotransmitters like purines or neuropeptides almost always involved in the plurichemical transmission and chemical coding of the autonomous nervous system.

Consequentially, this missing evidence for vesicular activity together with the fact that all wAPC cell bodies seemed to be in a very narrow contact to the surrounding neural meshwork perfectly fits with results of other findings. So we propose, that the nerve fibers contacting the wAPC correspond to the finely myelinated axons with a more electron-lucent matrix and a large number of neurofilaments but small in diameter and without any synaptic vesicles shown by Novotny^28^ and that they also may correspond to the sensory neurons in the dorsal root ganglia marked by retrograde tracing but showing none of the staining for known sensory neuropeptide neurotransmitters described by Kurkowski^29^. We suggest that the contact the wAPC membrane builds up to the axonal membrane is similar to that already shown for axonal contacts to lymphocytes^6^,^7^,^28^, so it might be in the range of nanometers. These narrow contact of the wAPC combined with our finding that the neural meshwork always only covers the cell body and does not follow any membranous extension opens up another interesting possibility in that cell body and membranous extensions of the wAPC may function as different domains regarding the innervation whereby we propose a sensory role for the neural meshwork surrounding the cell body of the wAPC.

So presuming a sensory role for the wAPC it is now necessary to address the functional aspect for this neurally hard-wired organization. As we can see, not every antigen presenting cell marked by the surface markers used is innervated by a neural meshwork. This taken together with the fact that it has not been possible to define a subset of cells always expressing a marker combination typical for resident or migratory antigen presenting cells in other mammals – because this differentiation of resident versus migratory through surface marker expression is still missing for rat antigen presenting cells – actually makes it difficult to make a statement about the function of the wAPC. However with our results regarding the dynamics or plasticity of the system through MAP2 and axonal growth cone staining we have to assume that the innervation of the wAPC we see here in fact is dynamic, not static and may depend on immunological activity of the innervated wAPC, taking into consideration that in a more general aspect, plasticity of innervation in mammalian lymph nodes has been shown by other groups in a different context^13^,^27^,^30^. This would foreground a migratory origin of the wAPC in that antigen presenting cells continuously coming in from the periphery build up neural contacts to sense their immunological status to the central nervous system, whereas the other and not innervated antigen presenting cells we identified would belong to the resident population of the lymph node. But as nature always uses many different pathways, we cannot exclude the alternative, that also some of the resident cells are innervated as a wAPC occupying other functions and sensing other immunological aspects. Interestingly, combining both alternatives would be a good explanation for the wired organization in that through contacts with single cells – resident also as migratory – the central nervous system would get the possibility to locate immunological events more precisely. Anyway with the method of immunohistochemical staining it is only possible to see a “snapshot”, so it will be of high interest to establish functional models for verifying that assumptions.

Lastely, as only two of the classical three known types of antigen presenting cells seem to be candidates for the wAPC, somebody may ask why B-cells as the third class of that cell type are not innervated. As B-cells also T-cells are not innervated, so the contact made by the nerve fibers seem to be specific only for two types of antigen presenting cells (namely dendritic cells and macrophages) and not for lymphocytes. So that contact may be restricted to antigen presenting cells of myeloid origin, taking into consideration that also a lymphoid origin of dendritic cells has been postulated. Moreover, the neural meshwork is exclusively found in a specific region (Paracortex), it seems not to be specific for a cell type, though most immune cells are concentrated in separate regions but also can be found dispersed through the rest of the lymph nodes tissue, where no single wAPC occurs. And as the B-cell gets their full activation through the second signal by a T-cell also in the innervated paracortical region, these wired nerval organization if sensory may be for sensing immunological events for orchestrating and even guiding lymphocytes to their respective antigen presenting counterpart.

This leads to a fascinating assumption in that it may be the answer to the unsolved question how especially T-cells can find one specific and fitting dendritic cell within billions of cells in a short time frame necessary for an adaptive immune response.

Whereas the completion of the maturation of the B-cell in the germinal center and subsequent affinity maturation through somatic hypermutation eventually does not need control by the nervous system because it is highly regulated within the follicle through a selective process.

## Conclusion

So with this work, we show for the first time ever a systematic and abundant specific and narrow contact between single and not endothel-associated nerve fibers and single cells with APC character via a “wired” cell enclosing neural mesh in lymph nodes of the rat which revealed a multitude of innervated cells grouped in a specific area that has even not been supposed until today.

These wAPC showing: (1) a narrow cell covering contact, (2) signs for neural plasticity and (3) a missing sympathetic and therefore probably not efferent innervation strongly indicate directed informational contact to single cells, a phenomenon often seen in mammalian sensory systems. So we suggest these wAPC to be sensory cells of an afferent pathway, postulated also by other groups and among other being the distal receptive part of the already traced pseudounipolar neurons and their peripheral axon with dendritic character^11^,^12^,^28^,^29^. With this system it would not only be possible for the nervous system to get topographical information about the location of immune threats, it may also be an approach for solving the question how the T-cells find their antigen presenting counterpart, in that the nervous system could guide these two cell-types being the central part of adaptive immune responses. It will be of great importance to find out the type of contact these wAPC build up to the nerval structures covering them as to handle the major questions concerning plasticity or static organization of the system, the origin and fate of the Wapc’s and to a great extent, what they “hear” and to whom they “talk”.

## Materials and Methods

### Immunohistochemistry/Immunofluorescence

Surgical explanted and without further fixation procedures directly snap frozen superficial cervical lymph nodes (LN) and brains (control tissue) of untreated and traced (see below “Tracer application”) Sprague-Dawley rats (250–280 g) were purchased from Harlan Laboratories GmbH (The Netherlands). Surgical explanted and without further fixation procedures directly snap frozen superficial cervical lymph nodes (LN) and brains (control tissue) of athymic Nude-Foxn1^nu^ Mice (NMRI) were purchased from Harlan Laboratories GmbH (The Netherlands).

All experimental procedures were approved by the DEC-Consult Animal Ethics Committee, Utrecht, The Netherlands, number HAR-0005 according to all applicable rules, laws and regulations.

Human normal lymph node samples (healty, 60 years old donor) were purchased as frozen tissue slides (Cat No.: T1234161) from the BioChain Institute, Inc. (BioCat. GmbH, Heidelberg, Germany).

All human tissue samples were collected with informed consents from the donors and their relatives, or collected under the FDA Guidance document issued on April 25, 2006 entitled “Guidance on Informed Consent for *In Vitro* Diagnostic Device Studies Using Leftover Human Specimens That Are Not Individually Identifiable” as delinked tissues which do not require informed consent from the donor, complying with all requirements set forth by Guidelines for Writing Informed Consent Document, the Office of Human Subjects Research (OHSR), National Institutes of Health (NIH). U.S. Department of Health and Human Services (DHHS).

20 μm cryo-sections were prepared with a Leica CM1850 cryostat, dried for 20 min. at room temperature and fixed in cold Aceton Methanol (1:1). Tissue sections were blocked with Super Block (ScyTek Laboratories (Logan, Utha, USA). Primary antibodies were rabbit anti-MAP2 (polyclonal) (1:500; SYSY, Göttingen, Germany), rabbit anti-synaptophysin (SP11) (1:200; DCS, Hamburg, Germany), mouse anti-CD11c (8A2) (1:100; AB-online, Aachen, Germany), mouse anti-CD11b/c (OX42) (1:500; Abcam plc, Cambridge, UK), mouse anti-CD103 (OX62) (1:100; Abcam plc, Cambridge, UK), mouse anti-SIRPalpha (OX41) (1:100; Abcam plc, Cambridge, UK), mouse anti-MHC II (OX6) (1:500; Abcam plc, Cambridge, UK), mouse anti-CD4 (W3/25) (1:100; Abcam plc, Cambridge, UK), mouse anti-CD68 (ED1) (1:100; Abcam plc, Cambridge, UK), mouse anti-CD90 (OX7) (1:100; Abcam plc, Cambridge, UK), mouse anti-Growth Cone (2G13) (1:5; Abcam plc, Cambridge, UK), mouse anti-Podoplanin (LF3(B7)D5B3) (1:100; Acris Inc., Herford, Germany), mouse anti-RECA-1 (HIS52) (1:50; Covalab SAS, France), rabbit anti-CD3 (SP7), (1:200; DCS, Hamburg, Germany), rabbit anti-S100B (polyclonal) (1:200; Boster Biological Technology, Fremont, USA), rabbit anti-S100B (polyclonal) (1:200; AB-online, Aachen, Germany) and mouse anti-NF (SMI312) (1:1000; BioLegend, San Diego, USA).

Primary antibody incubation for 60 min., followed by 15 min. incubation of secondary antibodies, goat anti-rabbit-DyLight 549 (1:700; DCS, Hamburg, Germany), goat anti-rabbit-Alexa 488 (1:700; DCS, Hamburg, Germany), goat anti-mouse-DyLight 549 (1:700; DCS, Hamburg, Germany), goat anti-mouse-Alexa 488 (1:700; DCS, Hamburg, Germany). Slides were mounted using Vectashield Hard Set mounting medium with DAPI (Vector Laboratories, Burlingame, CA, USA).

To avoid cross-reaction, primary antibodies used in multicolour staining were conjugated with: Cy3 (Cy3 Fast Conjugation Kit/Abcam plc, Cambridge, UK), FITC (FITC Fast Conjugation Kit/Abcam plc, Cambridge, UK), HiLyteFluor 647 (AnaTag HiLyte Fluor 647/ANASPEC, Inc., Fremont, USA) and HiLyteFluor 488 (AnaTag HiLyte Fluor 488/ANASPEC, Inc., Fremont, USA).

Every antibody staining was established and validated on appropriated control tissue, e.g. brain.

### Tracer application

5 Sprague-Dawley rats (250–280 g) were anesthestized with pentobarbital sodium (20 mg/kg, i.p.). A total volume of 1 μl of 30% Fluoro-Ruby (Merck KGaA, Darmstadt, Germany) in distilled water was injected into the superior cervical ganglion. After survival for 7 and 14 days, the animals were killed and the ipsilateral cervical lymph nodes were removed and snap frozen.

## Additional Information

**How to cite this article**: Wülfing, C. and Günther, H. S. Dendritic cells and macrophages neurally hard-wired in the lymph node. *Sci. Rep*. **5**, 16866; doi: 10.1038/srep16866 (2015).

## Supplementary Material

Supplementary Information
