# Supplementary material for: Dendritic cells and macrophages neurally hard-wired in the lymph node
Source: Sci Rep. 2015 Nov 19;5:16866. doi: 10.1038/srep16866 (PMC4652329; doi:10.1038/srep16866)
Supplement: Supplementary Information [file srep16866-s1.pdf]

Title:

Dendritic cells and macrophages neurally hard-wired in the lymph node.

Authors:

Clemens Wuelfing[1], Hauke S. Guenther[1]

Affiliations:

[1]Group for interdisciplinary neurobiology and immunology, Biozentrum Grindel, University of Hamburg

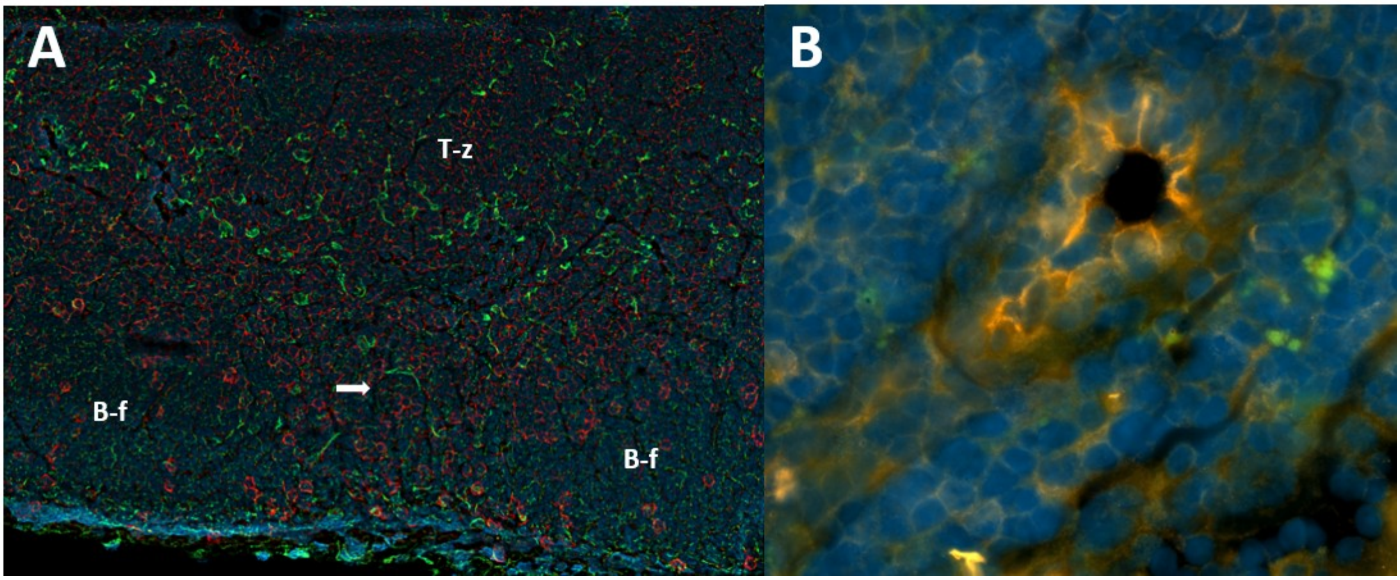

__Supplement Fig. S1 wAPC are located in the T-cell enriched zone:__ *Superficial cervical lymph nodes of DS rats stained with monoclonal anti-neurofilament (green) and monoclonal anti-CD3 (red) in Image A and monoclonal anti-endothelial RECA-1 (orange) in Image B. Image A as a 3D reconstruction (z-stack) shows a clear homing of the wAPC in the T-cell enriched area and sparse signals in the extrafollicular zone (white arrow) between two B-cell follicles which miss any NF signal (20 fold). Image B shows a high endothelial venule in the T-cell enriched zone with no wAPC in close contact (100 fold). T-z – T-cell enriched zone / B-f – B-cell follicle.*

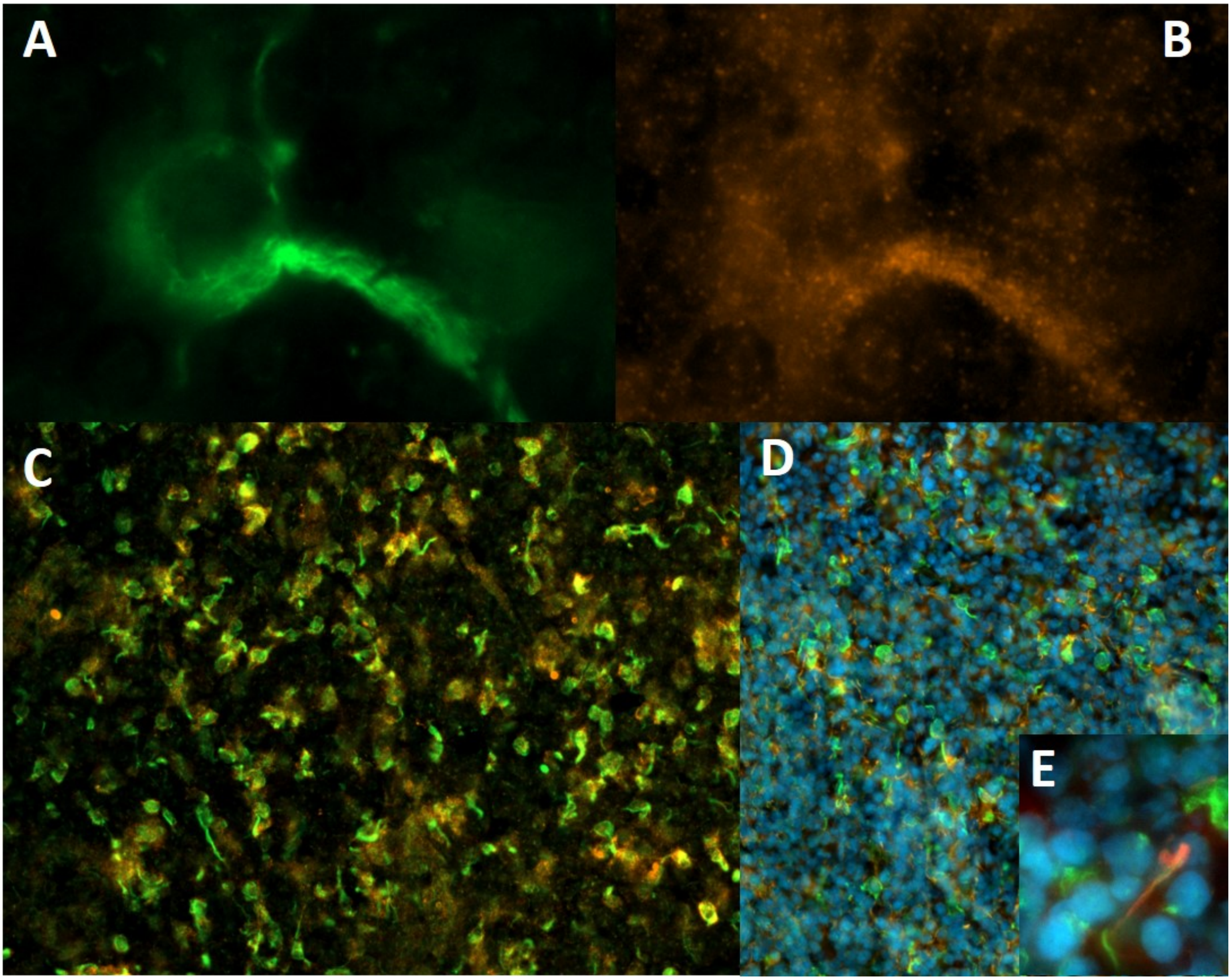

__Supplement Fig. S2 wAPC indicating plasticity with MAP2 and axonal growth cones:__ *Superficial cervical lymph nodes of DS rats stained with monoclonal anti-neurofilament (green) and polyclonal MAP2 (orange) in Image B and C or anti-growth cone 2G13p (orange / red) in Image D and E. Image A and B show one wAPC reached by a single nerve fiber and covered by the filamentous neurofilament signal and the granular and dotted MAP2 signal respectively (100 fold). The merged Image C confirmed the colocalisation of both neural markers around the wAPC in the lymph node T-cell enriched zone (40 fold). No colocalisation of MAP2 was detected in axonal structures in the medullary region, which is not shown here. Image D shows the staining for axonal growth cone in the wAPC area (40 fold), with Image E as a magnification out of Image D showing a potential candidate for building a dynamic growth cone (100 fold).*

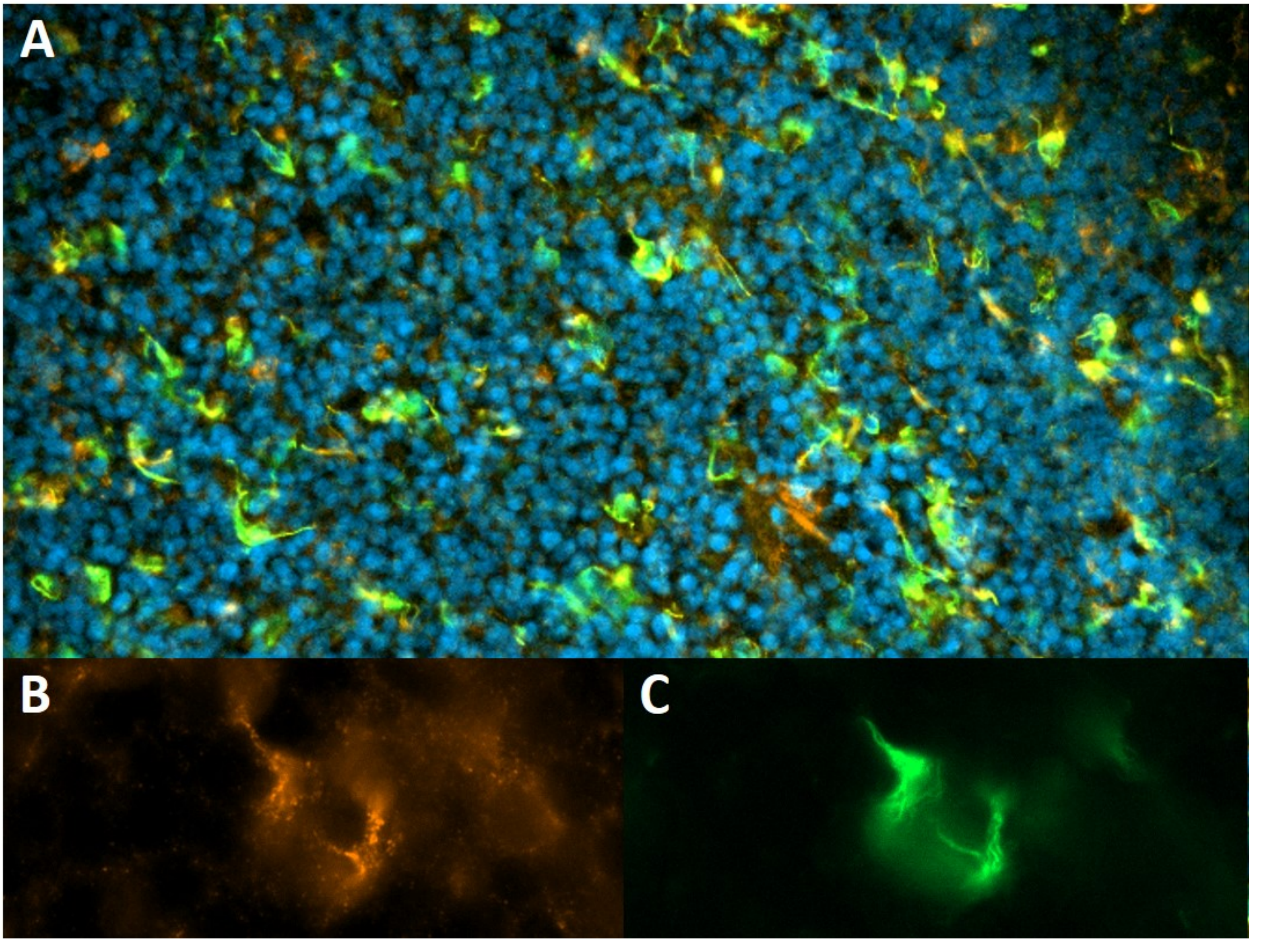

Supplement Fig. S3 SIRPα – wAPC marker or neural origin?: *Superficial cervical lymph nodes of DS rats stained with monoclonal anti-neurofilament (green) and monoclonal anti-SIRPα (orange). The staining pattern of SIRPα is very similar to that of neurofilament. Image B and C (both 100 fold) show one wAPC where the more granular SIRPα signal resamples the filamentary neurofilament signal. Image A (40 fold) shows the colocalization of neurofilament and SIRPα.*

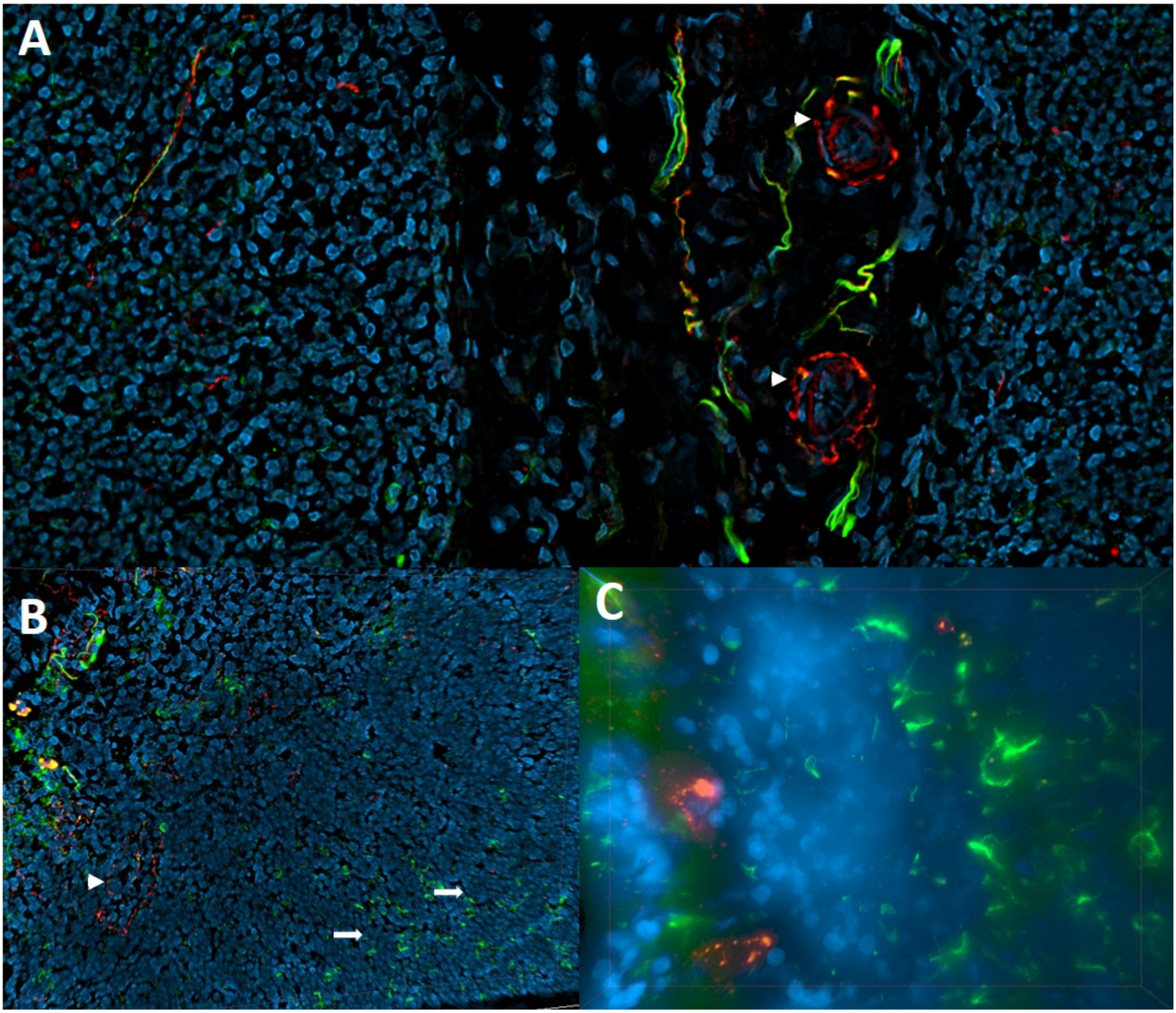

<u>**Supplement Fig. S4 wAPC and the missing sympathetic link:**</u> *Superficial cervical lymph nodes of DS rats stained with monoclonal anti-neurofilament (green) and polyclonal synaptophysin (red) in Image A and B and Fluoro-Ruby (red) in Image C. Image A shows synaptophysin signals consequently arranged around blood vessels (white arrowheads)(20 fold). Colocalisation between neurofilament and synaptophysin was partially found at some filamentous axonal structures in the medulla. In Image B signals for synaptophysin are again detected presumably around blood vessels (white arrowheads), in partial colocalisation with green neurofilament signals but not at the wAPC in the T-cell enriched zone (white arrows), (20 fold, 3D reconstruction of z-stack). Image C shows the red Fluoro-Ruby-Tracer signal marking sympathetic efferences at distinct cells than the wAPC (green / 100 fold).*

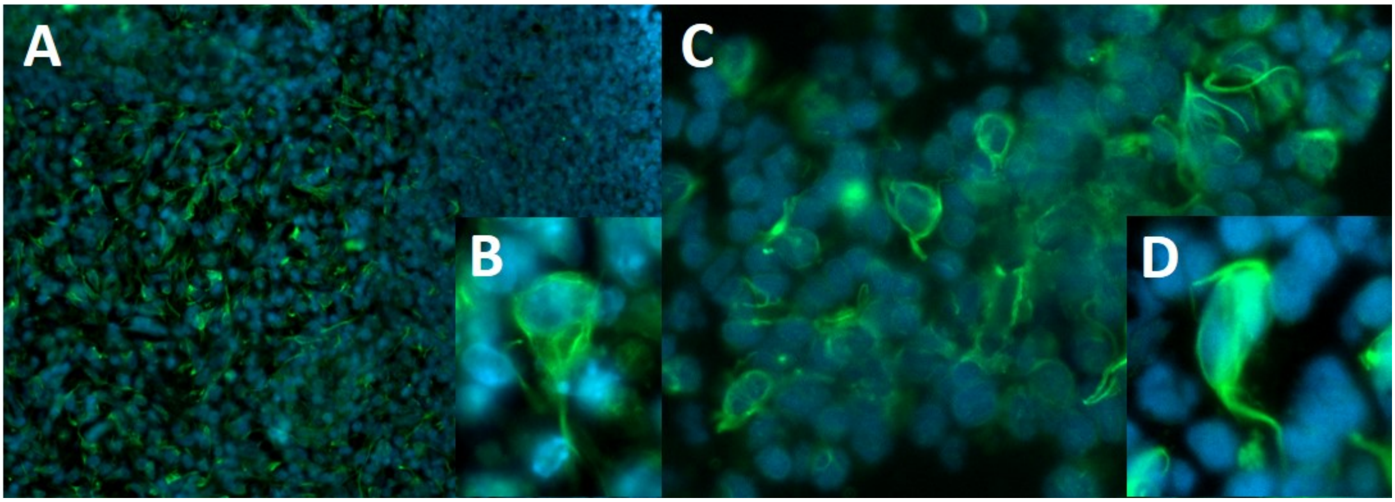

<u>Supplement Fig. S5 wAPC in mice and men:</u> *Lymph node from a T-cell deficient mice (Image A and B) and of a healthy human 60 years old male (Image C and D) stained with monoclonal anti-neurofilament (green). The wAPC show the same morphology and density like in rats, and are also detectable in the T-cell lacking "T-cell zones" of nude mice.*
